# Motion acquisition of gait characteristics one week after total hip arthroplasty: a factor analysis

**DOI:** 10.1007/s00402-024-05245-1

**Published:** 2024-03-14

**Authors:** Andrea Cattaneo, Anna Ghidotti, Francesco Catellani, Gennaro Fiorentino, Andrea Vitali, Daniele Regazzoni, Caterina Rizzi, Emilio Bombardieri

**Affiliations:** 1 Department of Information Management Engineering and Production Engineering, University of Bergamo, Via Galvani, 2, Dalmine, BG Italy; 2 Humanitas Gavazzeni, Bergamo, Italy

**Keywords:** Total hip replacement, Gait analysis, Factor analysis, Motion capture, Recovery of function, Surgical approach

## Abstract

**Introduction:**

Clinical gait analysis can be used to evaluate the recovery process of patients undergoing total hip arthroplasty (THA). The postoperative walking patterns of these patients can be significantly influenced by the choice of surgical approach, as each procedure alters distinct anatomical structures. The aim of this study is twofold. The first objective is to develop a gait model to describe the change in ambulation one week after THA. The secondary goal is to describe the differences associated with the surgical approach.

**Materials and methods:**

Thirty-six patients undergoing THA with lateral (n = 9), anterior (n = 15), and posterior (n = 12) approaches were included in the study. Walking before and 7 days after surgery was recorded using a markerless motion capture system. Exploratory Factor Analysis (EFA), a data reduction technique, condensed 21 spatiotemporal gait parameters to a smaller set of dominant variables. The EFA-derived gait domains were utilized to study post-surgical gait variations and to compare the post-surgical gait among the three groups.

**Results:**

Four distinct gait domains were identified. The most pronounced variation one week after surgery is in the Rhythm (gait cycle time: $+32.9\mathrm{\%}$), followed by Postural control (step width: $+27.0\mathrm{\%}$), Phases (stance time: $+11.0\%$), and *Pace* (stride length: − $9.3\%$). In postsurgical walking, *Phases* is statistically significantly different in patients operated with the posterior approach compared to lateral (p-value = 0.017) and anterior (p-value = 0.002) approaches. Furthermore, stance time in the posterior approach group is significantly lower than in healthy individuals (p-value < 0.001).

**Conclusions:**

This study identified a four-component gait model specific to THA patients. The results showed that patients after THA have longer stride time but shorter stride length, wider base of support, and longer stance time, although the posterior group had a statistically significant shorter stance time than the others. The findings of this research have the potential to simplify the reporting of gait outcomes, reduce redundancy, and inform targeted interventions in regards to specific gait domains.

**Supplementary Information:**

The online version contains supplementary material available at 10.1007/s00402-024-05245-1.

## Introduction

Total hip arthroplasty (THA) is the surgical procedure advised to treat end-stages osteoarthritis (OA). There are several surgical approaches for THA and these include, with some variations, the lateral, anterior and posterior approaches [1, 2]. An analysis of current practices based on national joint registries indicates that the majority of surgeons often chose the posterior approach, followed by the lateral and anterior one. The posterior approach preserves the abductors and flexors, but the external rotators need to be detached to gain access to the hip joint and the sciatic nerve may be compromised. The lateral approach requires division of the anterior third of the gluteal muscles and detachment of the proximal third of the vastus lateralis, with a constant risk of postoperative limping. The direct anterior approach is gaining popularity as a muscle sparing technique in which hip joint exposure is achieved following an inter-muscular plane between sartroius, rectus femoris, iliopsoas and tensor fasciae latae [3–5]. Each surgical method presents advantages and limitations and the most effective approach remains controversial in terms of surgeons learning curve, intra and post-operative complications, hospitalization time and patient subjective satisfaction [6, 7]. The primary hypothesis of this study was that different surgical approach alters different anatomical structures and influence patients’ healing process and recovery, as suggested by Mantovani et al. [8].

Evaluation of clinical outcomes for THA as mitigation of pain, improved quality of life and restoration of hip function are based on surveys, such as WOMAC, Harris Hip Score, SF-36, PROM-10 and HOOS [9, 10]. Nevertheless, these surveys are subjective and may not be sensitive enough to detect minor changes [11, 12]. In recent decades, there has been an increasing interest in the relationship between surgical approaches and gait biomechanics. A number of studies have assessed gait changes in patients after THA, with some suggesting the integration of clinical gait analysis for recovery measurement [7, 13–15]. Gait analysis provides quantitative data, crucial for studying the mechanisms of gait changes and evaluating the effectiveness of interventions, offering valuable insights into predicting hospital stay length and post-operative rehabilitation [14]. Most studies have focused on walking performance at 3, 6, and 12 months after surgery, providing limited information on the duration of hospitalization after surgery [17, 18]. However, gait analysis conducted seven days after THA can assist in developing timely rehabilitation strategies, which has become increasingly important in light of the reduced hospitalization period following surgery from weeks to days [19]. Shibuya et al. [16] attempted to address this need by investigating gait at 5 days post-surgery. However, regarding spatiotemporal parameters, they only evaluated gait speed using a stopwatch, neglecting other valuable variables obtainable with motion capture (mocap) systems [20]. In this context, the main aim of this paper is to fill this gap by developing spatiotemporal models of the short-term gait variation in patients undergoing THA. For this purpose, exploratory factor analysis (EFA) was performed on a dataset of gait spatiotemporal parameters acquired before and 7 days after surgery through a mocap system. The proposed model enables the identification of the gait domains affected by the surgery and measures the magnitude of change a week after surgery. The second aim is to describe the gait differences associated with the three surgical approaches (lateral, anterior, and posterior).

## Methods

### Experimental protocol

For this study, an ad-hoc protocol was created and approved by the institutional review board of Humanitas Gavazzeni (Bergamo, Italy). The protocol involved acquiring data from patients before and 7 days after surgery to assess short-term recovery. Gait data were collected using the Microsoft Kinect v2, a motion-sensing input device that combines a color camera and a depth camera to track and capture body movements [21]. Microsoft Kinect sensors have demonstrated significant potential in orthopaedics, particularly for obtaining spatiotemporal parameters of gait [22, 23]. The accuracy of the sensors, in terms of measuring depth, is reported to be under 2 mm in the central viewing cone, which makes them suitable for the study [24]. Two devices were placed diagonally opposite to each other, about 6 m apart, in the rehabilitation gym of the clinic [25]. Patients were asked to walk along a straight path at a self-selected comfortable speed for three runs. Three recordings per patient session were performed, as Maynard et al. suggested that a minimum of three gait cycles should be averaged to overcome the effects of stride-to-stride variability [26]. All patients followed the same rehabilitation protocol. From the first post-operative day, permissive weight bearing was allowed. Passive and active range of motion of the operated joint was assisted by a therapist, as postural changes from lied or sitting posture to a standing position.

### Patients’ enrollment

Patients who were admitted to the Orthopaedics and Traumatology wards of Humanitas Gavazzeni, and who underwent THA consecutively, using any access approach were enrolled for the study. The recruitment period spanned from January 2019 to February 2020, at which point it was temporarily halted due to the COVID-19 pandemic restrictions. The enrollment process resumed in May 2022 and continued until October 2022. All surgical procedures were carried out by senior consultant surgeons, who selected the most appropriate approach based on their expertise and experience. Therefore, the type of surgical approach (lateral, anterior, and posterior) did not depend on the study design, but only on the clinical situation and the choice of the individual operator. Patients with neurological diseases and neuromotor disorders were excluded from the study. Bearers of contralateral hip replacement, ipsilateral and contralateral knee replacement or subjects who had previously undergone to prosthetic revision surgeries were excluded. Enrolled patients had to be able to walk with or without cane support. All patients were well-informed about the noninvasive protocol and signed informed consent.

### Gait parameters

The study utilized raw depth maps obtained from Microsoft Kinect v2 to generate a virtual representation of the patient's gait using iPiSoft software. Spatiotemporal characteristics of the gait pattern were then calculated, including conventional variables such as gait cycle duration, stance time, swing time, double support time, step length, stride length, step width, peak swing velocity, and average gait speed. The variables were normalized based on the patient's height and gait cycle duration. The parameters that were dependent on the limb side were divided into ipsilateral and contralateral legs. The study also evaluated gait asymmetries using the difference between the parameters of the affected side and the unaffected side. To mitigate the impact of noise, the mean feature value derived from the three recordings per session was utilized for the analysis, as suggested by Maynard et al. [26].

### Statistical analysis

Gait analysis enables the collection of numerous variables, each providing valuable information. However, large dimensionality presents analytical challenges. Interpretation complexity can escalate, and there is an increased risk of Type I error (false positives) due to the higher likelihood of obtaining statistically significant results by chance. To address these challenges, EFA is a valuable technique. EFA groups highly correlated variables into common factors, reducing the dataset's dimensionality to overcome the aforementioned challenges.

The statistical analysis comprised three phases: (1) data preprocessing and data quality assessment; (2) EFA for dimensionality reduction and identification of dominant variables; (3) analysis of gait characteristics and gait changes. All computations were performed in Python with the following packages: numpy, pandas, scipy, and factor-analyzer.

### Data preprocessing

To ensure proper input data quality for EFA, the collected data were preprocessed as follows. First, the dataset was normalized by dividing each variable by the maximum absolute value of the feature. This process maintained the sparsity of the data without shifting or centering it. Second, each parameter was individually tested for EFA eligibility through the Kaiser–Meyer–Olkin (KMO) test. Variables not deemed suitable for EFA (KMO < 0.5) were excluded. The suitability of the resulting dataset was then checked through two statistical tests: (a) Bartlett’s test of homoscedasticity with $\alpha \le 0.05$ and (b) KMO test on the entire dataset with a minimum threshold of 0.5, as suggested by Hair et al. [27].

### Exploratory factor analysis (EFA)

EFA is a statistical method employed in multivariate analysis to examine the underlying structure of a set of observed variables and identify the latent factors that contribute to the observed patterns. In gait analysis, EFA reduces a large set of observed gait parameters into unobservable gait domains that explain the covariation among observed variables, revealing the underlying structure and inherent patterns within the gait data.

In this study, EFA with promax rotation was performed. The number of factors included in the model was determined based on Kaiser's criterion (eigenvalues > 1) [28]. Factor loadings ≥ 0.5 were considered to be relevant. The resulting model was named “Model A”. Initially, EFA was independently applied to preoperative and postoperative data. However, as both analyses revealed a similar factor structure, the decision was made to formulate the final model using the entire dataset for improved interpretability.

Although latent factors are effective in representing the dataset's structure, interpreting their raw values can be challenging since they do not express directly observable quantities. To improve interpretability, each factor was reduced to its “dominant variable”, which is defined as the parameter with the highest absolute factor loading. This streamlined version was referred to as "Model B".

### Gait characteristics analysis

After the development of gait models, a comprehensive statistical analysis was conducted to explore the extent to which gait characteristics are affected by THA surgery in the short-term. Cohen's $d$ was used to quantify the difference between preoperative and postoperative measures of latent factors ("Model A"). This standardized effect size expresses the variation between two means in standard deviation units. As it is dimensionless, it allows for the comparison of factors with diverse units of measurement. Following established guidelines, $d$ values of 0.2 to 0.5 were considered as small, 0.5 to 0.8 as medium, and greater than 0.8 as large [29]. To analyze the change in the dominant variables (“Model B”) the percentage of change was chosen for enhanced interpretability. Uncertainty was expressed as standard error calculated by bootstrapping with 10,000 resamples. For postoperative gait characteristics, the assumptions of parametric tests were not met, therefore the Kruskall-Wallis test and the Wilcoxon signed-rank test with α = 0.05 were used to investigate differences between surgical approaches. Statistically significant differences were further examined using the post hoc Conover's test with Holm-Bonferroni adjustment.

## Results

### Patients’ characteristics

**Fig. 1:**
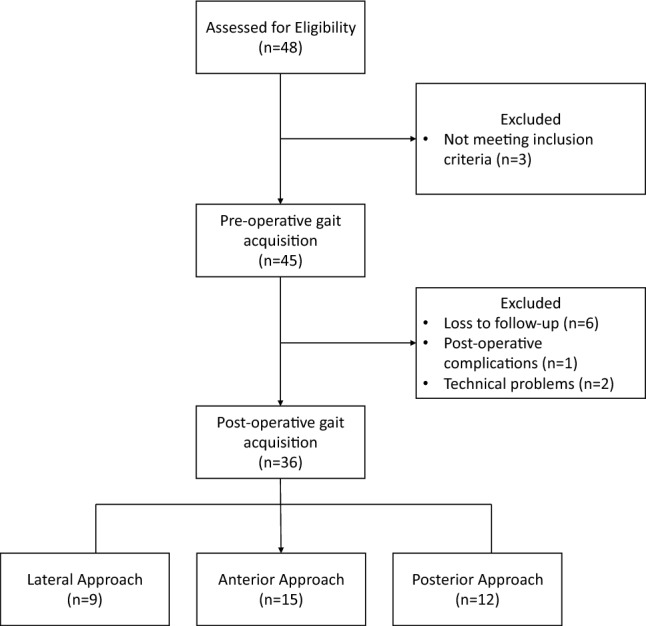
CONSORT diagram representing the flow of patients through the study. Forty-eight patients were assessed for eligibility. Three patients were excluded due to a contralateral hip prosthesis. Hence, forty-five patients were acquired by motion capture before the surgery. Six of these patients were lost in the follow-up, one had post-operative complications and two were excluded due to technical problems. Hence, thirty-six subjects performed the post-operative gait analysis

Forty-eight patients who underwent THA at the Orthopaedics and Traumatology unit of Humanitas Gavazzeni Hospital were enrolled in this study. The flow of subjects through the study is shown in the CONSORT diagram in Fig. 1.

Patients had a mean age of 67.9 years (range: 45–81). Sixteen patients were women and the remaining twenty patients were men (Table 1). All patients had monolateral (22 right, 14 left) hip OA grade II or III according to the Tonnis classification. Age and height were not significantly different between the groups (one-way ANOVA), nor were sex and side affected (G-test for independence). Surgeons determined the approach based solely on patient conditions, rather than study goals, resulting in varying group sizes.

**Table 1:** Demographic characteristics of subjects

	Lateral approach	Anterior approach	Posterior approach	p-value
Age (years)	70.4 [56–79]	68.9 [51–81]	64.2 [45–81]	0.269
Sex (F/M)	5/4	7/8	4/8	0.579
Height (cm)	169.4 [155–185]	168.1 [155–185]	168.0 [159–185]	0.932
Side affected (R/L)	5/4	10/5	8/4	0.837

Mean and range are given where relevant. P-values of one-way ANOVA analysis for numerical variables and G-test of independence for categorical variables are shown

### Feature selection

**Fig. 2:**
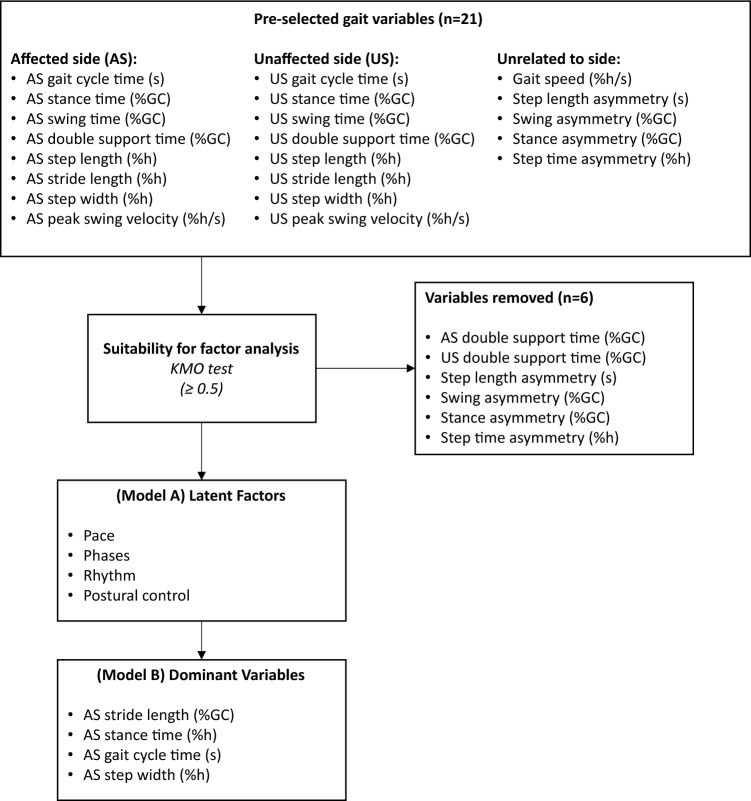
Flowchart of the development process of the two short-term gait change models. Abbreviations: affected side (AS), unaffected side (US), %h percent of height, %GC percent of gait cycle, Kaiser–Meyer–Olkin (KMO)

The analysis started with 21 gait spatiotemporal parameters reported in Table 2. The KMO test showed that 6 features were not suitable for EFA, thus they were removed (Fig. 2). The final dataset consisted of 15 variables and was deemed suitable for factor analysis, as indicated by a KMO test score of 0.729 and a p-value of < 0.001 in Barlett's test of sphericity.

**Table 2:** Changes of gait characteristics before (PRE) and 7 days after THA surgery (POST)

	Variable	PRE	POST	Effect size
Affected side (AS)	Gait cycle time (s)	1.309 (0.031)	1.715 (0.079)	1.131 (0.175)
	Stance time (%GC)	0.523 (0.019)	0.571 (0.025)	0.367 (0.157)
	Swing time (%GC)	0.478 (0.018)	0.450 (0.020)	− 0.247 (0.139)
	Double support time (%GC)	0.259 (0.020)	0.311 (0.027)	0.367 (0.113)
	Step length (%h)	0.293 (0.011)	0.275 (0.011)	− 0.283 (0.132)
	Stride length (%h)	0.604 (0.021)	0.540 (0.021)	− 0.519 (0.147)
	Step width (%h)	0.079 (0.003)	0.096 (0.004)	0.852 (0.196)
	Peak swing velocity (%h/s)	1.462 (0.046)	1.207 (0.059)	− 0.803 (0.156)
Unaffected side (US)	Gait cycle time (s)	1.277 (0.028)	1.736 (0.075)	1.358 (0.176)
	Stance time (%GC)	0.531 (0.019)	0.597 (0.024)	0.518 (0.166)
	Swing time (%GC)	0.474 (0.019)	0.417 (0.021)	− 0.473 (0.151)
	Double support time (%GC)	0.263 (0.021)	0.306 (0.026)	0.299 (0.100)
	Step length (%h)	0.279 (0.011)	0.246 (0.013)	− 0.472 (0.144)
	Stride length (%h)	0.594 (0.018)	0.545 (0.021)	− 0.425 (0.139)
	Step width (%h)	0.078 (0.004)	0.096 (0.003)	0.853 (0.293)
	Peak swing velocity (%h/s)	1.477 (0.042)	1.236 (0.056)	− 0.813 (0.175)
Unrelated to side	Gait speed (%h/s)	0.456 (0.023)	0.325 (0.021)	− 1.002 (0.201)
	Gait cycle time asymmetry (s)	0.032 (0.014)	− 0.021 (0.034)	− 0.336 (0.177)
	Swing time asymmetry (%GC)	0.005 (0.009)	0.033 (0.014)	0.397 (0.202)
	Stance time asymmetry (%GC)	− 0.007 (0.009)	− 0.025 (0.015)	− 0.245 (0.198)
	Step length asymmetry (%h)	0.014 (0.008)	0.029 (0.010)	0.273 (0.180)

Mean values and standard error (in parenthesis) are reported. Parameters are grouped based on the affected and unaffected sides. Effect sizes are represented as Cohen’s d. *%h* percent of height, *%GC* percent of gait cycle

### Gait models

**Fig. 3:**
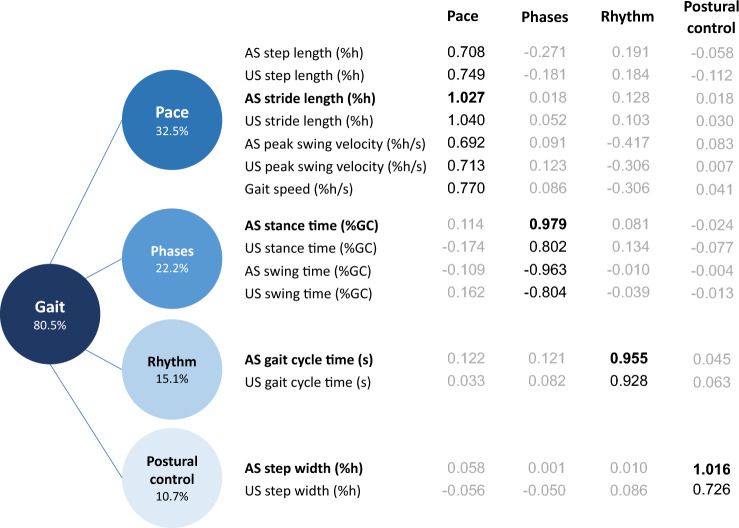
Model of the gait in patients one week after THA surgical intervention. Factor analysis of the selected 19 spatiotemporal parameters resulted in four orthogonal gait domains. Factor loadings considered relevant (≥ 0.5) are shown in black. The dominant variables of each latent factor are bolded. The values in the circles represent the proportion of total variance explained by each domain. Affected side (AS), unaffected side (US), %h percent of height, %GC percent of gait cycle

The 15 selected spatiotemporal parameters were included in “Model A”. EFA yielded four orthogonal factors accounting for 80.5% of the total variance. Factor loadings, which indicate the degree of association between the observed variables and the latent factors, are presented in Fig. 3. In light of them, factors were labeled as Pace (32.5% of total variance), Phases (22.2%), Rhythm (15.1%), and Postural control (10.7%), adhering to the gait domains classification of Gouelle et al. [30]. The factor loadings obtained solely from preoperative and postoperative data exhibited a consistent structure (see Supplementary Material—S1). Notably, no cross-loadings were observed, ensuring a clear delineation of the latent factors identified. The dominant variables for each factor were all related to the affected side, specifically stride length (representative of Pace), stance time (Phases), gait cycle time (Rhythm), and step width (Postural control). These variables compose the simplified “Model B”, effectively reducing the number of gait variables to collect from fifteen to the four most relevant and representative.

### Short-term gait performance after THA

**Fig. 4:**
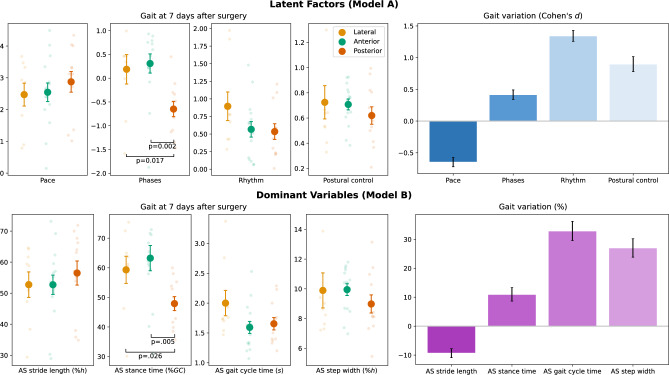
Plots illustrating gait traits at 7 days after THA surgery and the variation with preoperative conditions. The results are presented across the gait domains (Model A) and through the change in the respective dominant variables (Model B). The marked dots represent the mean value, the shaded dots the individual patient values, and the error bars represent the standard error estimated by bootstrapping with 10,000 resamples. In the postoperative walk, statistically significant differences were detected across approaches in the latent factor "Phases" and in the corresponding dominant variable "AS stance time." Horizontal black bars show the p-values of the post hoc study. Abbreviations: affected side (AS), percent of height (%h), percentage of gait cycle (%GC)

The gait domain that exhibited the most pronounced variation was Rhythm ($d=1.34\pm 0.17$) (Fig. 4). Postural Control followed, but still with a large effect size ($d=0.90\pm 0.23$), while Pace showed a negative effect size ($d=-0.65\pm 0.14$), indicating a reduction in walking speed post-surgery. Phases demonstrated a only small effect size ($d=0.42\pm 0.15)$. In light of 'Model B', it can be stated that the major gait changes found 7 days after THA are:

- Longer time to complete strides (AS gait cycle time: $+32.9\%\pm 6.7$)
- Wider base of support (AS step width: $+27.0\%\pm 6.3$)
- Longer foot contact to the ground (AS stance time: $+11.0\%\pm 4.6$)
- Shorter stride length (AS stride length: $-9.3\%\pm 3.1$)

While the observed pattern of variation in gait characteristics remains consistent across all surgical approaches, significance is identified when analyzing post-surgical walking in both “Model A” (p-value = 0.004, Kruskal–Wallis test) and “Model B” (p-value = 0.007). Specifically, relative stance time was found to be lower with the posterior approach ($47.8\%GC\pm 2.3$) compared to the lateral ($59.3\%GC\pm 4.6$) and anterior ($63.2\%GC\pm 4.3$) approaches. Notably, stance time for the posterior approach group is also significantly different from the golden ratio, a value associated with the walking of healthy individuals (p-value < 0.001, Wilcoxon test). In contrast, patients operated with lateral and anterior approaches exhibit a stance time compatible with the reference value [31]. However, it should be noted that no firm conclusions should be drawn due to limited statistical power.

## Discussion

This study investigated the gait characteristics of patients who underwent THA one week after surgery, using EFA. Due to the increasing number of THA cases and escalating healthcare expenses, there is a pressing need to efficiently manage the process to enhance patient recovery, reduce complications, and shorten hospital stays [32]. In this context, clinical gait analysis can play a crucial role in identifying suitable candidates for early discharge and ensuring the quality of the recovery process. In literature, there are many studies investigating THA effectiveness in the mid and long term (3–12 months) [17, 18]. However, to the best of the author’s knowledge, this is the first attempt to investigate gait characteristics one week after THA.

A novel aspect of this work is the application of EFA to model the gait of THA patients. While motion capture systems allow for the measurement of numerous walking features during gait analysis, conducting statistical tests on all gait parameters poses a notable risk of false positives. To mitigate this, data reduction techniques, such as EFA, are employed to minimize information loss by reducing the number of features. This approach allows for a focused examination of the most pertinent factors. These reduction strategies have been applied to evaluate gait dysfunctions related to neurological disorders, including multiple sclerosis [33], Parkinson’s disease [20], and post-stroke hemiplegia [14, 34, 35]. Boekestein et al. [12] have also employed EFA to investigate the gait patterns of individuals with hip and knee OA and found that the factors loaded on cadence and stride length showed the greatest difference compared to the control group.

In the present research, EFA reduced the measured 21 variables to four factors, that can be reconducted to well-known gait domains [30]. “Model A” is composed of four latent factors: Pace, Phase, Rhythm, and Postural Control, which explain 80.5% of the total variance. The first factor, labeled Pace, represents the overall performance of walking and it is loaded on gait speed, peak swing velocity, step length and stride length of both affected and unaffected limbs [30]. Maximal gait speed can be a predictor of functional recovery after THA, with mid-long term studies showing improved speed; however, deficits were still present, when compared with healthy controls [17, 18]. The study revealed a post-surgery decline in mean gait speed ($d=-1.00\pm 0.20$) and a reduction in stride length, identified as a dominant variable ($d=-0.52\pm 0.15$). They both are linked to insufficient torque generation from the lower extremities, which could lead to dynamic instability, potentially increasing the risk of falling [36]. These findings are reflected by the medium variation of this domain after surgery ($d=-0.65\pm 0.14$). The second factor is named Phases and describes the different moments of walking, including stance and swing time. Gait showed a small variation in this domain ($d=0.42\pm 0.15)$. For this factor, the stance phase was selected as dominant variable. In healthy subjects, the ratio between the stance time and the gait cycle is equal to the inverse of the golden ratio ${\varphi }^{-1}\cong 61.8\%$ [31], which is an irrational number with specific harmonic iterative properties also found in other biological fields. This proportion can be disrupted by walking disorders [37]. The third factor is called Rhythm and reflects the patients' gait cycle, which is even the dominant variable. In this study, this domain is the most affected by THA in the short-term ($d=1.34\pm 0.17)$, possibly due to the (a) pain associated with the procedure and (b) muscle and soft tissue damage caused by the surgical procedure [6, 38]. The fourth factor, Postural control, is related to the step width, which is the dominant variable. In the present study, step width increased on both the affected and unaffected sides after surgery. This domain experienced a large variation ($d=0.90\pm 0.23)$ in the immediate postoperative, a behavior typically linked to compensatory patterns adopted to maintain balance and that is influenced by pain.

The investigation detected significant changes in gait characteristics seven days after surgery. This model allows an in-depth analysis of significant factors influencing gait patterns, facilitating the development of recovery methods that are more targeted. A comparative examination of the gait domains chosen sheds light on the differences between the three surgical approaches.The influence of the surgical approach on the surgery outcome is widely studied. Mantovani et al. have claimed that the choice of the surgical approach may influence postoperative gait [8]. However, there is much debate about which is the most effective solution [6]. The anterior approach has increased in popularity among surgeons and patients because it seems to offer better early outcomes in terms of pain, rehabilitation, and length of stay [39﻿]. However, as of today, no clear evidence emerged to support the superiority of any approach in the mid to long term [40]. The results of this study evidence statistically significant differences in Phases domain between the posterior approach group and both the lateral (p-value = 0.017) and anterior (p-value = 0.002) approach groups. This finding suggests that the surgical approach used may have an impact on gait within the first week after surgery. Patients who underwent the posterior approach exhibited a significant deviation from the golden ratio in their gait cycle to stance time ratio ($47.8\%\pm 2.3$, p-value < 0.001), while no significance was found with the lateral and anterior approaches. This result is supported by “Model B”, where there are statistically significant differences in stance time between the posterior approach group and both the lateral (p-value = 0.026) and anterior (p-value = 0.005) approach groups. This impairment could be the result of the damage of the hip-abductor muscle, which plays a significant role during stance, and is more likely to occur in the posterior approach [41]. Based on the results of this study, it appears that the surgical approach may affect the short-term recovery of THA patients in terms of gait patterns. By incorporating these findings into the decision-making process, clinicians will have the opportunity to make more informed decisions, potentially resulting in improved outcomes for patient recovery and hospital stays.

This work has some limitations. First, this is a single-center study that involved only European patients. Therefore, the findings may lack external validity in other regions and contexts. Second, the sample size is limited, thereby limiting the statistical power of some tests. However, the dataset's suitability for EFA was demonstrated to be acceptable. Moreover, the gait analysis was performed using markerless mocap devices, which may produce inaccurate measurements under certain circumstances, although technical precautions were made to limit this risk. Finally, patients' gait was recorded in the presence of researchers and physicians, a condition that may have led them to consciously or subconsciously modify their spontaneous gait pattern [42]. This could potentially affect ecological validity, although participants underwent familiarization sessions to mitigate context-induced alterations.

## Conclusions

This study presented a four-component gait model tailored to individuals undergoing THA. It allows for condensing a large set of spatiotemporal gait parameters into a smaller number of variables. By focusing solely on these four dominant variables, the most important gait characteristics can be captured with minimal information loss. This reduces the burden of gait acquisition and computation, making gait analysis in clinical settings more accessible.

The model identified the major changes in gait characteristics experienced at 7 days postoperatively: longer time to stride but shorter stride length, wider support base, and prolonged ground contact. The posterior approach group had a significantly shorter stance time compared to both the other approaches and the optimal reference value. This suggests that the posterior approach may impair gait Phases shortly after surgery. Additional studies are needed to confirm this claim.

### Supplementary Information

Below is the link to the electronic supplementary material.

Supplementary file1 (PDF 170 KB)

## Data Availability

The raw datasets underlying this study are not publicly available due to privacy concerns outlined in the research protocol. Anonymized and aggregated data are available upon request from the corresponding author.
