# Supplementary material for: Motion acquisition of gait characteristics one week after total hip arthroplasty: a factor analysis
Source: Arch Orthop Trauma Surg. 2024 Mar 14;144(5):2347–56. doi: 10.1007/s00402-024-05245-1 (PMC11093841; doi:10.1007/s00402-024-05245-1)
Supplement: Supplementary file 1 — Supplementary file1 (PDF 170 KB) [file 402_2024_5245_MOESM1_ESM.pdf]

S1 – Supplementary Information

Article Title: Motion acquisition of gait characteristics one week after total hip arthroplasty: A factor analysis
Journal Name: Archives of Orthopaedic and Trauma Surgery
Authors: Andrea Cattaneo[1] · Anna Ghidotti[1] · Francesco Catellani[2] · Gennaro Fiorentino[2] · Andrea Vitali[1] · Daniele Regazzoni[1] · Caterina Rizzi[1] · Emilio Bombardieri[2]
Affiliations: [1]Department of Information Management Engineering and Production Engineering, University of Bergamo, Italy · [2]Humanitas Gavazzeni, Bergamo, Italy

Table S1 Factor loadings resulting from exploratory factor analyses performed separately on preoperative and postoperative gait parameters. Factor loadings considered relevant (≥0.5) are shown in black. Abbreviations: affected side (AS), unaffected side (US), %h percent of height, %GC percent of gait cycle.

Factor label	Variable	Pre-operative				Post-operative			
Pace	AS step length (%h)	0.768	0.112	0.243	-0.003	0.578	-0.383	0.087	-0.117
	US step length (%h)	0.671	0.226	0.235	-0.041	0.769	-0.119	0.181	-0.186
	AS stride length (%h)	0.955	-0.024	0.098	0.092	1.028	-0.002	0.152	-0.065
	US stride length (%h)	1.000	-0.097	0.140	0.046	1.014	0.027	0.079	-0.042
	AS peak swing velocity (%h/s)	0.725	-0.031	-0.360	-0.119	0.776	0.084	-0.323	0.215
	US peak swing velocity (%h/s)	0.725	-0.036	-0.174	-0.064	0.833	0.216	-0.245	0.076
	Gait speed (%h/s)	0.774	-0.043	-0.346	0.079	0.913	0.044	-0.148	0.086
Phases	AS stance time (%GC)	0.045	-0.961	-0.076	0.011	0.171	0.998	0.099	-0.101
	US stance time (%GC)	-0.018	-0.983	0.121	-0.024	-0.292	0.724	0.056	-0.148
	AS swing time (%GC)	-0.036	0.962	0.010	0.021	-0.185	-0.947	-0.064	-0.046
	US swing time (%GC)	0.001	0.985	-0.121	-0.019	0.265	-0.721	0.029	-0.009
Rhythm	AS gait cycle time (s)	0.053	-0.050	0.939	-0.056	0.085	0.137	0.910	0.129
	US gait cycle time (s)	0.000	-0.055	0.963	0.021	-0.087	0.057	0.901	0.096
Postural control	AS step width (%h)	0.041	-0.046	-0.048	0.999	0.074	-0.052	0.103	0.884
	AS step width (%h)	0.002	0.051	0.003	0.869	-0.094	-0.043	0.071	0.719
